# Effect of nursing care based on goal-oriented mind mapping model on the prognosis of patients with severe brain injury

**DOI:** 10.1097/MD.0000000000039896

**Published:** 2024-10-11

**Authors:** Jingxue Zhang, Xin'an Jiao, Xianjun Ma, Ruizhao Yu, Jing Pan, Meiling Yuan, Shuaihui Wang, Changbao Hua, Hongmei Pan

**Affiliations:** a Department of Critical Care Medicine, Qinghe County Central Hospital, Xingtai, Hebei Province, China; b Department of Orthopedic, Qinghe County Central Hospital, Xingtai, Hebei Province, China; c Nursing Department, Qinghe County Central Hospital, Xingtai, Hebei Province, China.

**Keywords:** goal oriented, mind mapping, prognosis, quality of life, severe brain injury

## Abstract

The objective of this study was to observe the effect of nursing care based on goal-oriented mind-mapping on the prognosis of patients with severe brain injury. Clinical data of 116 patients with severe brain injury admitted to Qinghe County Central Hospital between March 2021 and August 2023 were retrospectively analyzed. Based on the nursing mode the patients received, they were divided into an Observation group (n = 58, patients received nursing based on the goal-oriented mind mapping mode) and a Control group (n = 58, patients received routine care). Data on length of hospital stay, complications, functional recovery, cerebral oxygen metabolism, and quality of life scores of the 2 groups were collected and analyzed. The length of hospital stay of the Observation group was shorter than that of the Control group (*P* < .05). The total incidence of complications in the Observation group was lower than that in the Control group (*P* < .05). After intervention, neurological function, cerebral oxygen metabolism indicators, and quality of life of the 2 groups improved significantly compared with those before the intervention; furthermore, the neurological function and cerebral oxygen metabolism indexes of the Observation group were better than those of the Control group (*P* < .05). The nursing care based on goal-oriented mind-mapping model for patients with severe brain injury can effectively shorten the length of hospital stay, reduce the occurrence of prognostic complications, and improve the recovery of neurological and limb motor functions, and ultimately achieve the goal of improving the quality of life.

## 1. Introduction

With the development of industries such as construction, transportation, and extreme sports, the incidence of brain injury is on the rise.^[1]^ It has an acute onset, progresses rapidly over a short period of time, and has a poor prognosis. It is one of the leading causes of mortality and disability worldwide.^[2,3]^ Brain injury can be classified as mild, moderate, severe, or extremely severe according to the Glasgow Coma Scale (GCS). Patients with severe brain injury (GCS score ≤ 8 points) have rapidly changing conditions and are usually complex to treat, which can result in varying degrees of physical disability and mental impairment. During the treatment, complications such as pulmonary infection and deep vein thrombosis (DVT) are prone to occur, which may have a negative impact on patients’ prognosis.^[4]^

Nursing plays an important role in reducing secondary injuries and improving prognosis in patients with severe brain injury.^[5]^ Although routine nursing emphasizes the care of patients with severe traumatic brain injury, it lacks systematic, targeted, and comprehensive care. The goal-oriented approach is to solve problems in the patient’s rehabilitation process by setting goals, so as to improve the prognosis. It has good application effects in critically ill patients and can effectively improve the patient’s survival rate and prognosis. However, the rehabilitation cycle of patients with severe brain injury is longer and the nursing care requirements are different at each stage,^[6]^ which leads to a large amount of nursing content and is difficult to memorize. In this case, the use of visual mind maps that focus on keywords and stimulate divergent thinking can further optimize the nursing process. Research has shown that the mind mapping model can effectively enhance nurses’ nursing competencies.^[7]^ The combination of goal orientation and mind mapping can make the nursing care of patients with severe brain injury more targeted, comprehensive, and efficient.

Our hospital implemented nursing care based on goal-oriented mind mapping model for patients with severe brain injury and found that it can effectively improve the prognosis of patients. In this study, we reviewed the clinical data of the patients received the nursing care based on goal-oriented mind mapping model and analyzed the treatment status of the patients, aiming to provide a reference for relevant clinical nursing care.

## 2. Materials and methods

### 2.1. Patients

Clinical data of patients with severe brain injury admitted to Qinghe County Central Hospital from March 2021 to August 2023 were retrospectively analyzed. The study included 76 males and 40 females. Inclusion criteria were (a) patients who met the diagnostic criteria for severe brain injury and underwent successful neurosurgery; (b) patients with a GCS score of 3 to 8 points; and (c) patients with complete clinical data. Exclusion criteria were (a) patients with severe liver or kidney dysfunction, or mental illness; (b) patients participating in other studies during the same period; (c) patients complicated with brain metastases or tumors; or (d) pregnant women: based on the nursing mode the patients received, they were divided into an Observation group (patients received nursing based on the goal-oriented mind mapping mode) and a Control group (patients received routine care).

### 2.2. Intervention methods

**Figure 1.**
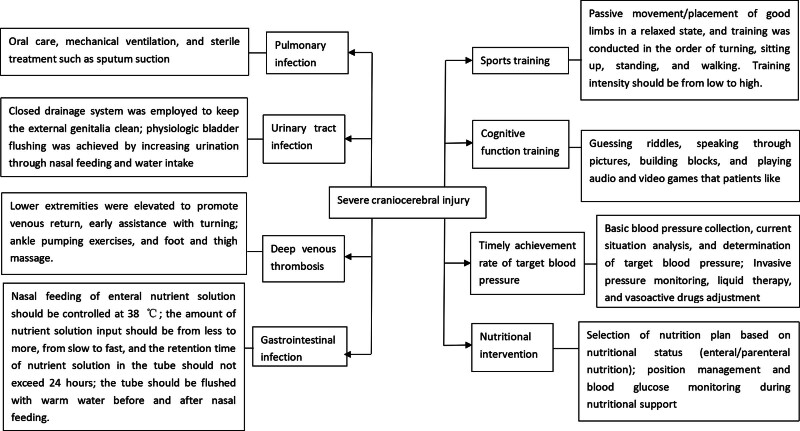
Mind map of goal-oriented nursing model.

(1) Patients in the Control group received routine nursing care, including health education, maintaining a clean and tidy ward environment, close monitoring of patients’ vital signs, strict implementation of aseptic procedures, and symptomatic treatment.
(2) Patients in the Observation group received nursing care based on goal-oriented mind mapping model. A nursing team was formed, led by the head nurse and responsible nurse, and the attending physician was consulted. The team first reviewed the literature and combined it with clinical practice to analyze the factors affecting the prognosis of severe brain injury, and then developed a nursing plan based on goal-oriented mind mapping model (Fig. 1). After the nursing plan was determined, the responsible nurse would provide content training to the team members. The nursing care based on goal-oriented mind mapping model was as follows: (1) Infection management. (A) Pulmonary infection management. Sodium bicarbonate/saline was given for oral care after tracheotomy, comatose patients were given suction treatment for oral secretions, the tracheal cannula opening was covered with sterile gauze, and mechanical ventilation and suctioning operations were performed with strict adherence to asepsis. (B) Urinary tract infection management. Closed drainage systems were employed to keep the external genitalia clean; physiologic bladder flushing was achieved by increasing urination through nasal feeding and water intake. (C) Gastrointestinal infection management. Nasal feeding of enteral nutrient solution should be controlled at 38 °C; the amount of nutrient solution input should be from less to more, from slow to fast, and the retention time of nutrient solution in the tube should not exceed 24 hours; the tube should be flushed with warm water before and after nasal feeding. (2) Deep vein thrombosis management. Lower extremities were elevated to promote venous return, early assistance with turning, ankle pumping exercises, and foot and thigh massage. (3) Sports training. Passive movement of the limbs in a relaxed state, and training was conducted in the order of turning up, sitting up, standing up, and walking. Training intensity should be from low to high. (4) Cognitive function training. Guessing riddles, speaking through pictures, building blocks, and playing audio and video games that patients like. (5) Timely achievement rate of target blood pressure. Basic blood pressure collection, current situation analysis, and determination of target blood pressure. Invasive pressure monitoring, liquid therapy, and vasoactive drugs adjustment. (6) Nutritional intervention. Selection of nutrition plan based on nutritional status (enteral/parenteral nutrition); position management and blood glucose monitoring during nutritional support.

### 2.3. Collection indicators

(1) Length of hospital stay. (2) Complications, including pulmonary infection, gastrointestinal infection, urinary tract infection, and deep vein thrombosis. (3) Brain oxygen metabolism, including levels of jugular venous oxygen saturation (SjvO2) and cerebral extraction of oxygen (CEO2). (4) Functional recovery. The National Institutes of Health Stroke Scale (NIHSS) and Fugl-Meyer Assessment (FMA) scales were used. The total NIHSS score is 42 points, with higher scores indicating poorer neurological function. The total FMA score is 100 points, with higher scores indicting better limb motor function. (5) Quality of life. The 36-item Short Form Health Survey was used. The 36-item Short Form Health Survey score includes 8 dimensions, with a total score of 100 points; the higher the score, the higher the quality of life in the relevant dimension.

### 2.4. Statistical analysis

Data were analyzed using SPSS version 26.0 (IBM Corp, Armonk, NY). Counting data were expressed as percentages (%), and chi-square test was used for comparison between the 2 groups. Data of normal distribution were expressed as mean ± standard deviation, and *t* test was used for comparison between the 2 groups; paired sample *t* test was used for intra-comparison, and independent sample *t* test was used for intergroup comparison. *P* < .05 was considered statistically significant.

## 3. Results

**Figure 2.**
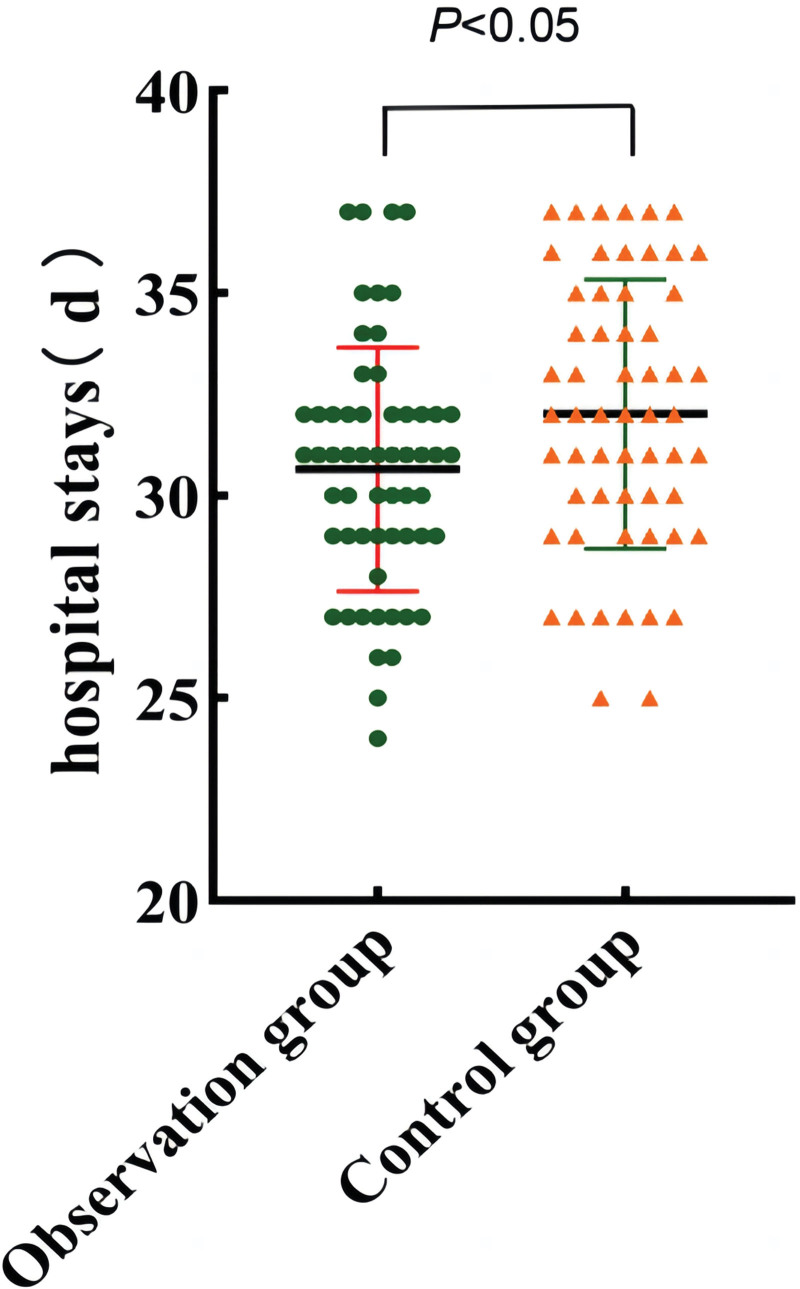
Comparison of length of hospital stay between the 2 groups.

A total of 116 patients with severe brain injury were included in this study with 58 patients in each group. There was no statistically significant difference in baseline characteristics between the 2 groups (*P* > .05) (Table 1). The length of hospital stay of the Observation group (30.66 ± 3.00 days) was significantly shorter than that of the Control group (32.02 ± 3.33 days) (*P* < .05) (Fig. 2). The incidence of complications in the Observation group (10.34%) was significantly lower than that in the Control group (32.76 %) (*P* < .05) (Table 2). After intervention, the NIHSS scores of the 2 groups were lower than those before intervention, and the Observation group was significantly lower than the Control group (*P* < .05); the FMA scores of the 2 groups were significantly higher than those before intervention, and the Observation group was significantly higher than the Control group (*P* < .05) (Table 3). After the intervention, the SjvO2 levels were significantly lower in both groups than before the intervention, and the levels were lower in the Observation group (*P* < .05); the CEO2 levels were significantly higher in both groups than before the intervention, and the levels were higher in the Observation group (*P* < .05) (Table 4). After the intervention, the scores of physical vitality, physical pain, emotional function, physiological intelligence, mental health, overall health, social function, and physiological function in both groups were significantly higher than those before the intervention, and the scores in the Observation group were higher (*P* < .05) (Table 5).

**Table 1:** Comparison of baseline characteristics between the 2 groups.

Characteristics	Category	Observation group (n = 58)	Control group (n = 58)	*χ*^2^/*Z*/*t* value	*P* value
Gender	Male	39 (67.24)	37 (63.79)	0.153	.696
	Female	19 (32.76)	21 (36.21)		
Age	≥60 years	23 (39.66)	26 (44.83)	0.318	.573
	<60 years	35 (60.34)	32 (55.17)		
Injury type	Cerebral contusion and laceration	17 (29.31)	15 (25.86)	‐0.440	.660
	Intracranial hematoma	33 (56.90)	34 (58.62)		
	Subdural hematoma	8 (13.79)	9 (15.52)		
Trauma cause	Traffic injury	36 (62.07)	33 (56.90)	-0.563	.574
	Fall injury	16 (27.59)	18 (31.03)		
	Other	6 (10.34)	7 (12.07)		
Diabetes	Yes	5 (8.62)	7 (12.07)	0.372	.542
	No	53 (91.38)	51 (87.93)		
Hypertension	Yes	4 (6.90)	6 (10.34)	0.438	.508
	No	54 (93.10)	52 (89.66)		
Educational level	Junior high school and below	21 (36.21)	19 (32.76)	‐0.532	.595
	High school/technical secondary school	13 (22.41)	12 (20.69)		
	College or above	24 (41.38)	27 (46.55)		
BMI (kg/m^2^)		22.41 ± 3.22	23.40 ± 3.14	‐1.668	.098
GCS mark (points)		5.29 ± 1.58	5.22 ± 1.59	0.234	.815
Time from injury to treatment (minute)		4.567 ± 1.20	4.59 ± 1.22	0.381	.704
Operation time (minute)		70.22 ± 8.61	71.61 ± 9.17	‐0.841	.402

GCS = Glasgow Coma Scale.

**Table 2:** Comparison of complications between the 2 groups.

Group	Pulmonary infection	Gastrointestinal infection	Urinary tract infection	Deep vein thrombosis	Overall incidence
Observation group (n = 58)	2 (3.45)	2 (3.45)	1 (1.72)	1 (1.72)	6 (10.34)
Control group (n = 58)	6 (10.34)	5 (8.62)	4 (6.90)	4 (6.90)	19 (32.76)
*χ^2^* value					8.617
*P* value					.003

**Table 3:** Comparison of NIHSS and FMA scores between the 2 groups.

Group	NIHSS		FMA	
	Pre-intervention	Post-intervention	Pre-intervention	Post-intervention
Observation group (n = 58)	24.19 ± 2.61	10.83 ± 1.34*	60.38 ± 5.13	83.16 ± 7.05*
Control group (n = 58)	24.74 ± 2.67	16.62 ± 1.75*	61.40 ± 5.01	75.53 ± 7.25*
*t* value	‐1.124	-20.052	-1.080	5.742
*P* value	.263	.000	.282	.000

*Note*: Compared with the same group before treatment.

FMA = Fugl-Meyer Assessment, NIHSS = National Institutes of Health Stroke Scale.

* *P* < .05.

**Table 4:** Comparison of serum SjvO2 and CEO2 levels between the 2 groups.

Group	SjvO2 (%)		CEO2 (%)	
	Pre-intervention	Post-intervention	Pre-intervention	Post-intervention
Observation group (n = 58)	73.40 ± 8.47	59.95 ± 6.28*	19.70 ± 2.31	26.70 ± 3.09*
Control group (n = 58)	72.28 ± 9.41	64.02 ± 6.56*	19.47 ± 2.39	23.40 ± 3.31*
*t* value	0.674	-3.413	0.529	5.562
*P* value	.502	.001	.598	<.001

*Note*: Compared with the same group before treatment.

CEO2 = cerebral extraction of oxygen, SjvO2 = jugular venous oxygen saturation.

* *P* < .05.

**Table 5:** Comparison of quality of life scores between the 2 groups.

Item	Pre-intervention		*t* value	*P* value	Post-intervention		*t* value	*P* value
	Observation group (n = 58)	Control group (n = 58)			Observation group (n = 58)	Control group (n = 58)		
Physical vitality	53.09 ± 4.29	54.12 ± 4.20	-1.312	.192	67.47 ± 6.07*	61.71 ± 5.44*	5.382	<.001
Somatic pain	53.86 ± 4.35	54.09 ± 4.27	-0.28	.780	67.98 ± 5.10*	63.93 ± 5.02*	4.314	<.001
Affective function	51.34 ± 4.72	52.36 ± 4.90	-1.138	.257	64.02 ± 5.13*	58.83 ± 5.17*	5.423	<.001
Physiological function	51.41 ± 4.28	52.02 ± 4.25	-0.762	.447	68.05 ± 5.14*	63.93 ± 6.00*	3.974	<.001
Mental health	54.33 ± 4.16	54.93 ± 4.10	-0.787	.433	66.05 ± 5.09*	62.57 ± 5.97*	3.382	<.001
General health	51.29 ± 4.06	51.79 ± 4.99	-0.592	.555	68.53 ± 5.01*	63.74 ± 5.94*	4.696	<.001
Social function	54.07 ± 4.86	54.78 ± 4.76	-0.791	.431	66.78 ± 4.98*	62.41 ± 5.12*	4.649	<.001
Physiological function	51.47 ± 4.63	52.22 ± 4.48	-0.897	.371	67.07 ± 4.99*	62.55 ± 5.98*	4.417	

*Note*: Compared with the same group before treatment.

* *P* < .05.

## 4. Discussion

The study showed that implementing a nursing care based on goal-oriented mind mapping model for patients with severe brain injury can effectively shorten their length of hospital stay, reduce the occurrence of prognostic complications, and improve the recovery of neurological and limb motor functions.

Brain injury refers to open or closed brain injury caused by various external violent injuries, such as traffic accidents, high-altitude falls, and firearm injuries, which directly or indirectly affect the head.^[8]^ Patients may experience a series of symptoms such as cerebral hemorrhage, cerebral contusion, and edema.^[9]^ Severe brain injury is a serious and complicated condition, and surgery can quickly relieve the irreversible brain damage that occurs after the brain tissue is compressed.^[10]^ With the maturity of surgical techniques and the improvement of surgical instrument precision, the clinical treatment effect for brain injuries has been improved.^[8,10]^ However, postoperative infections can easily occur and seriously affect patient recovery. Research has shown that the incidence of postoperative pulmonary infections after severe brain injury surgery ranges from 19.9% to 33.5%.^[11]^ Surgical time, mechanical ventilation time, and tracheotomy are risk factors of lung infection in patients with traumatic brain injury.^[12,13]^ After surgery, patient’s respiratory cilia movement is weakened, making it difficult for respiratory secretions to be discharged normally, resulting in inhalation of oral secretions and increasing the risk of pulmonary infection. Tracheotomy is beneficial for the care of the patient’s respiratory tract, but it can lead to damage to the tracheal and bronchial mucosa as well as thick sputum, which can easily lead to lung infection.^[14]^ Therefore, in case of pulmonary infection, the oral cavity and related ducts must be cleaned and aseptic operation procedures must be strictly followed during surgery. Multiple intestinal infections are a common type of hospital infection in traumatic brain injury and the main cause of death in patients with severe traumatic brain injury.^[15]^ Patients with brain injury are in a state of hypercatabolism, and they are unable to eat normally for a period of time. Long-term and heavy use of antibiotics can cause gastrointestinal dysfunction and bacterial imbalance in the intestinal tract, leading to gastrointestinal infections. Appropriate nutritional support can not only improve the patient’s nutritional status, but also promote the recovery of the patient’s tissue function.^[16]^ Research has shown that nutrient solution temperature can cause gastrointestinal infections.^[17]^ Gastrointestinal infections can affect the body’s absorption and utilization of nutrients, as well as affect the body’s water electrolytes and acid–base balance. Therefore, when providing enteral nutrition to patients, it is necessary to maintain the appropriate temperature of the nutrient solution and follow the order of administering enteral nutrition from less to more and from slower to faster in order to improve the patient’s intestinal tolerance. Urinary tract infection is also a hospital-acquired infection in patients with severe brain injury,^[18]^ but it is often overlooked and affects the prognosis of patients. Patients with severe traumatic brain injury usually require an indwelling urinary catheter because of impaired consciousness and surgical procedures. Fungus around the urethra enters the bladder through the catheter and multiplies in the bladder, forming an abscess through the kidneys. When the abscess ruptures, pus enters the bladder and causes urinary tract infections.^[17,18]^ Once a urinary tract infection occurs in patients with severe brain injuries, their inability to urinate can also affect the duration of indwelling catheters and exacerbate the difficulty of treatment. Therefore, it is necessary to attach great importance to the prevention of this type of infection. Not only should aseptic operation be performed, but the patient’s urethral orifice and vulva should also be kept clean, and the bladder can also be flushed by drinking water.

In addition to hospital-acquired infections, DVT is a common complication in patients with severe head injuries, with an incidence rate of up to 40%.^[19]^ Multiple injuries, lung infections, and long-term bed rest have been reported as risk factors for DVT.^[20]^ Patients with severe brain injury are in a low flow rate state because they need to stay in bed for treatment, which makes their limbs unable to move effectively. In addition, trauma leads to the entry of endothelial cells, activating the coagulation system and causing the body in a hypercoagulable state. Moreover, the use of dehydrating agents and intravenous injection of irritant drugs during the treatment can make patients highly susceptible to DVT. Therefore, measures such as assisting with turning and early exercise can be taken to reduce the occurrence of DVT, but patient tolerance should always be monitored to adjust the intervention content.

Routine nursing emphasizes the provision of high-quality services to patients with severe brain injury to reduce its occurrence.^[21]^ However, it is limited by the difference in nursing capabilities of medical staff, rapid changes in patients’ conditions, and heavy nursing workload, leading to a lack of systematic, comprehensive, and individualized nursing, which in turn affects the patients’ prognosis. The nursing care based on goal-oriented mind mapping model combines the advantages of both goal orientation and mind mapping. The goal-oriented approach develops personalized and systematic intervention strategies with the goal of postoperative rehabilitation for patients with severe brain injury, and develops intervention strategies for pulmonary infections, gastrointestinal infections, urinary tract infections, and DVT that are prone to occur in patients with severe brain injury, which can not only provide targeted and personalized nursing services for patients, but also enhance the ability of medical staff to identify and manage complications.^[22,23]^ Meanwhile, the large amount of nursing content can easily lead to boredom among medical staff and patients due to receiving too much information, which is not conducive to the smooth implementation of nursing content. However, mind mapping can process boring information into a graphic mode, and its procedural, systematic, and organizational nature can facilitate users to quickly understand and grasp its content. Visualizing information is also beneficial for patients and their families to understand and improve patient compliance with treatment. Functional rehabilitation training is also an important factor affecting patient prognosis when developing nursing care based on a goal-oriented mind mapping model. Patients with severe brain injury are in an ischemia and hypoxic state due to the sharp increase in intracranial pressure and local microcirculation disorder. According to the theory of brain plasticity and functional reorganization,^[24]^ early exercise and cognitive function training can accelerate the establishment of cerebral collateral circulation in patients, and ultimately improve and restore various body functions. Studies have shown that SjvO2 and CEO2 are closely related to the prognosis of patients with traumatic brain injury.^[25]^ An SjvO2 > 75% indicates an increase in cerebral oxygen supply/blood flow in patients with severe brain injury.^[26]^ CEO2 is a commonly used clinical indicator of cerebral oxygen supply, and a decrease in CEO2 indicates a decrease in cerebral oxygen metabolism.^[27]^ In terms of SjvO2 and CEO2 levels, SjvO2 decreased and CEO2 increased in both groups of patients after intervention, but the Observation group had lower SjvO2 and higher CEO2 after the intervention compared to the Control group, suggesting that the patients in the Observation group had a better prognosis.

Limitations of this study: This study retrospectively selected patients with severe brain injury as the research subjects and did not select mild to moderate patients for analysis. In addition, patients with severe brain injury may also experience mental disorders such as depression and anxiety. These negative emotions can also affect the patient’s enthusiasm for rehabilitation exercises and their prognosis. Therefore, psychological assistance measures are needed to promote treatment compliance and reduce patients’ negative emotions.

## 5. Conclusion

The nursing care based on goal-oriented mind mapping model for patients with severe brain injury can effectively shorten the length of hospital stay, reduce the occurrence of prognostic complications, and improve the recovery of neurological and limb motor functions, and ultimately achieve the goal of improving quality of life. These findings should be interpreted cautiously and prospective multicenter studies are required to further validate the findings.

## Author contributions

**Conceptualization:** Jingxue Zhang, Xin’an Jiao.

**Data curation:** Xianjun Ma, Jing Pan.

**Formal analysis:** Jing Pan, Shuaihui Wang.

**Investigation:** Ruizhao Yu, Meiling Yuan.

**Methodology:** Changbao Hua.

**Software:** Hongmei Pan.

**Writing – original draft:** Xin’an Jiao.

**Writing – review & editing:** Jingxue Zhang.
